# Uncertainties and contradictions experienced by women with COVID-19 during childbirth/birth and the postpartum period

**DOI:** 10.1590/0034-7167-2024-0236

**Published:** 2025-03-14

**Authors:** Maria Aparecida Baggio, Amanda Martins de Souza, Rosane Meire Munhak da Silva, Marli Terezinha Stein Backes, Adriana Zilly

**Affiliations:** I Universidade Estadual do Oeste do Paraná. Cascavel, Paraná, Brazil; II Universidade Estadual do Oeste do Paraná. Foz do Iguaçu, Paraná, Brazil; III Universidade Federal de Santa Catarina. Florianópolis, Santa Catarina, Brazil

**Keywords:** Women, Parturition, Postpartum Period, COVID-19, Obstetrics, Mujeres, Parto, Periodo Posparto, COVID-19, Obstetricia

## Abstract

**Objectives:**

to understand experiences and meanings attributed to childbirth/birth and postpartum by women affected by COVID-19.

**Methods:**

qualitative study in light of complexity, conducted through interviews with 15 women in university hospital reference for infected and high-risk pregnant women in tenth Health Region of Paraná, Brazil, between December 2021 and April 2022. Data were analyzed using thematic analysis.

**Results:**

childbirth/birth and postpartum experiences different from what was desired and planned, permeated by uncertainties, unpredictability and contradictions, especially regarding maternal and neonatal outcomes, were identified. Experiences full of meanings and emotions caused suffering and compromised mental health, particularly related to prematurity, newborn separation, absence of companion and isolation in COVID-19 unit. Mother-baby bond, self-care and breastfeeding damage, and antagonistic professional actions were observed, with a setback in obstetric practices.

**Final Considerations:**

there is a need to respect the rights guaranteed by law and designate professionals trained for maternal and child care.

## RESUMEN

**Objetivos:**

comprender las experiencias y significados atribuidos al parto/nacimiento y posparto por mujeres afectadas por COVID-19.

**Métodos:**

estudio cualitativo de complejidad, realizado mediante entrevistas a 15 mujeres en un hospital universitario referencia para embarazadas infectadas y de alto riesgo en la décima Región Sanitaria de Paraná, Brasil, entre diciembre/2021 y abril/2022. Los datos fueron analizados mediante análisis temático.

**Resultados:**

se identificaron experiencias de parto y posparto diferentes a lo deseado y planificado, permeadas por incertidumbres, imprevisibilidad y contradicciones, especialmente en lo que respecta a los resultados maternos y neonatales. Experiencias llenas de significados y emociones provocaron sufrimiento y comprometieron la salud mental, particularmente relacionadas con la prematuridad, el retiro del recién nacido, la ausencia de un acompañante y el aislamiento en una unidad COVID-19. Se encontraron daños en el vínculo madre-bebé, el autocuidado y la lactancia materna, y acciones profesionales antagónicas, con regresión a las prácticas obstétricas.

**Consideraciones Finales:**

se destaca la necesidad de respetar los derechos garantizados por la ley y designar profesionales calificados para la atención materno infantil.

## INTRODUCTION

The COVID-19 pandemic was declared by the World Health Organization (WHO) on March 11, 2020^(1)^, paralyzing economic and social life in the world and giving rise to a health catastrophe with an alarming death toll. A crisis was created, forcing human beings to reflect on and adapt their living and working habits to the pronounced condition. Human beings were confined to tiny spaces (whether to protect themselves or to treat themselves, when victims) and exposed to an uncertain fate^(2)^.

In the health sector, in order to deal with the pandemic in its different phases, it was necessary to adopt strategies for managing the care of people infected by the virus or not, by managers and professionals of services, globally. To this end, the dynamics of care in hospitals were reconfigured, with adaptation of physical structure and work processes, management of human resources, implementation of strategies to prevent infection by the SARS-CoV-2 virus and guarantee of professional and user safety, in accordance with national, regional and international recommendations. In particular, university hospitals were requested to offer beds in wards and Intensive Care Units (ICU) for people with the infection^(3)^.

Due to the pandemic, obstetric care in hospitals has also undergone adjustments. New demands have emerged and, consequently, new forms of care have been incorporated into services, whereas others have been reduced. The challenging control of the pandemic through protective measures has increased the challenges for healthcare professionals in providing care during childbirth^(4)^ and postpartum.

Care during these transitional periods requires intensive support for women by healthcare professionals. However, changes in the birth experience, concerns about transmission of the virus, delays in care, newborn (NB) separation from their mothers, restrictions on companions and difficulty in establishing contact with them were some of the common conditions in maternity wards that weakened care^(5)^.

A study conducted in the United States on women’s experiences during pregnancy, childbirth/birth and postpartum during the COVID-19 pandemic found that experiences varied based on geographic location, parity and support they had, especially emotional support. The pandemic affected women and their families, especially regarding expectations versus reality of the experiences they had^(6)^.

Given the above, the question was: what were the experiences and meanings attributed to childbirth/birth and postpartum by women affected by COVID-19?

## OBJECTIVES

To understand experiences and meanings attributed to childbirth/birth and postpartum by women affected by COVID-19.

## METHODS

### Ethical aspects

The study is part of a larger project entitled “Coping with COVID-19 and Maternal and Child Care”, which complied with Resolution 466/2012 of the Brazilian National Council for Health and Research Involving Human Beings. To ensure anonymity, participants were identified by the letter “W”, for woman, followed by Arabic numerals, according to the order of the interview (W1... M15).

### Theoretical-methodological framework

Complexity theory was the theoretical framework used^(2,7)^.

### Study design

This is a qualitative study, using the COnsolidated criteria for REporting Qualitative research (COREQ) as a guide to guide the research process report.

### Study setting

University hospital of Cascavel, Paraná, Brazil, reference for infected and high-risk pregnant women, of the tenth Health Region of Paraná.

### Methodological procedures

Fifteen women who gave birth while diagnosed with COVID-19 participated in the study. Participants were selected from a list of 97 pregnant women admitted to the study hospital with COVID-19 infection between March 2020 and March 2022, as reported by the epidemiological surveillance service. Women were selected by convenience, using inclusion criteria. Telephone contact was made with 47 women, of whom 15 agreed to participate. In addition, 22 declined; five could not be contacted (incorrect telephone number); the families of three women reported death after childbirth; and two did not speak Portuguese.

Women over 18 years of age who gave birth during the peak of SARS-CoV-2 infection in a public hospital, a reference for hospitalization of pregnant women with suspected or diagnosed COVID-19, regardless of gestational age, were included. Women who did not speak Portuguese, had physical or mental health problems that prevented their participation, and those who died were excluded.

### Data collection and organization

Upon acceptance to participate in the research, a date and time for the interview were scheduled, according to participants’ availability. Twelve interviews were conducted via voice call and two via video call, via the WhatsApp^®^ application, and one in person, at the study location, with an average duration of 30 minutes, with privacy guaranteed. Three pilot interviews were conducted, which were included in the study because they provided in-depth coverage of the topic at hand. There was no need to adjust the instrument.

The interviews were conducted by a nursing undergraduate student, trained and supervised by the researcher in charge, who has expertise in qualitative research. Data collection took place between December 2021 and April 2022, through individual interviews, guided by a semi-structured script, starting with the triggering questions: tell me about your experience of childbirth and postpartum. Comment on the experience of your child’s birth. Talk about the meanings of your experience.

The interviews were audio-recorded, transcribed and returned to participants via WhatsApp^®^ for corrections to transcribed content. No participant suggested corrections. The phenomenon of saturation allowed the interviews to be closed-ended.

### Data analysis

The data were analyzed using thematic analysis^(8)^, which consists of three stages. In the first stage, pre-analysis, the data was read to achieve completeness, representation, homogeneity and relevance, based on the proposed objective. In the second stage, material exploration, the data was classified, considering the significant expressions and/or words, according to their core meanings, to identify categories and subcategories. In the third stage, data processing and interpretation, the inferences and interpretation of analysis content were based on the theoretical framework adopted in this study^(2,7)^.

## RESULTS

The participating women accessed the hospital due to signs of labor, suspected or diagnosed COVID-19 and/or respiratory complications. As a high-risk referral hospital, the hospital admitted women for childbirth and/or treatment of COVID-19 complications from other municipalities, from another health region of Paraná, such as the municipality of Assis Chateaubriand, in the twentieth Health Region of Toledo.

Most of them were symptomatic, had preterm pregnancies, were multiparous, had a mean age of 32.9 years, and two had perinatal death as an outcome. Of the ten women who had a surgical delivery route, four expressed the desire for natural birth, and one of them had a plan for both delivery and birth. Some said they had been infected by their partner, but most did not know who to contact. Vaccines were not yet available to pregnant women at the time of the study (Chart I).

**Chart 1:** Characterization of women in relation to maternal, obstetric, COVID-19 data and origin, Cascavel, Paraná, Brazil, 2021

W	Age	GA	Parity in admission	Reason for admission	Symptoms at admission	Birth route	Origin	Period: childbirth and interview
W1	42	38	PIVAICII	L	Yes	Surgical	Cascavel	7 months and 5 days
W2	35	37	PIVNBIII	WB	Yes	Surgical	Catanduvas	6 months and 22 days
W3	25	34	PIIINBII	WB	Yes	Vaginal	Cafelândia	9 months and 14 days
W4	31	31	PIIAI	Complication	Yes	Surgical	Assis Chateaubriand	1 year, 5 months and 24 days
W5	32	34	PI	L	Yes	Vaginal	Cascavel	10 months and 23 days
W6	26	28	PI	L	No	Vaginal^*^	Cascavel	1 year, 3 months and 17 days
W7	36	40	PI	L	Yes§	Surgical#	Cascavel	10 months and 23 days
W8	26	32	PI	Complication	Yes§	Surgical	Capitão Leônidas Marques	1 year, 1 month and 3 days
W9	27	38	PIICI	WB	Yes	Surgical	Cascavel	8 months and 2 days
W10	43	31	PIVAINBII	Symptoms	Yes§	Surgical	Cascavel	1 year, 1 month and 22 days
W11	39	38	PIIAI	Symptoms	Yes§	Vaginal ^*^	Cascavel	1 year, 10 months, 10 days
W12	41	32	PVIIAIINBIV	Complication	Yes	Surgical	Cascavel	11 months and 15 days
W13	27	32	PIIICII	Complication	Yes	Surgical^*^	Cascavel	8 months and 11 days
W14	22	39	PI	L	No	Vaginal	Quedas do Iguaçu	1 year, 3 months and 20 days
W15	42	31	PIVAINBII	Symptoms	Yes§	Surgical	Cascavel	1 year, 2 months and 23 days

* *Perinatal death; #Childbirth and birth plan; §Positive partner; W - woman; GA - gestational age; P - pregnancy; NB - natural birth; A - abortion; C - cesarean section; L - labor; WB - water breaking.*

Aspects related to the meanings of childbirth and birth, postpartum and breastfeeding experiences and (lack of) healthcare allowed the organization of three categories of analysis.

### Giving meaning to the experience of childbirth and birth

It was possible to identify, in the experiences, the unexpected, the unpredictable and the uncertain related to childbirth and birth. The unexpected was linked to multiple conditions, namely: premature labor, triggered by COVID-19; labor, even if full-term, but during the COVID-19 pandemic; clinical-obstetric complications resulting from SARS-CoV-2 infection; the experience of unplanned childbirth (surgical or induced); the place of childbirth (surgical center or COVID-19 unit); birth outcome; among other conditions.

Some women had their pregnancies terminated by induction of labor or cesarean section. According to statements, most women wanted a natural birth, although they did not have a plan for labor and childbirth. However, most underwent surgery in a surgical center, described as improvised, with several professionals present, some showing anxiety. Two experienced labor in the COVID-19 unit and two in the emergency room screening. It was different from what they imagined, planned, or wanted.

[...] *I was so scared* [...] *for me, I was going there [surgical center], they were going to take my son out* [cesarean section] *and I was going to die [...] it was full of nurses, there were four, five nurses* [...] *doctor, all terrified, all anxious* [...]*, I thought, “He’s going to kill me here!”* [...] *it was an improvised room* [...] *I was afraid of losing him [baby] while I didn’t see him being born* [...] *that he was fine, I didn’t feel calm.* (W2)

*There were nurses from the COVID ward and the obstetrician came* [...] *she was born* [in the COVID-19 ward] [...] *the nurses were there with her and, when she was about to be born, the obstetrician came. When she entered the room, she went to do the examination and then her water broke* [...] *she said that she was going to be born right there in that room* [...]. (W14)

*I was in the ambulance, I waited for an hour until they* [hospital staff] *found a stretcher* [...] *I started bleeding* [...] *it started to hurt more, I had contractions* [...] *I had the baby down there* [screening] *premature, at 7 months* [...]. *She* [doctor] *grabbed the baby and ran out with him* [...] *she went to the neonatal ward* [ICU] *to try to intubate the baby* [...] *I don’t know what happened up there* [...] [the newborn died]. (W6)

The unpredictable and uncertain were related to women’s clinical-obstetric status and to maternal and neonatal responses after childbirth and birth. Living or dying was uncertain for mother and child. The finitude of human beings was on the agenda, bringing forth a universe of meanings. An experience of motherhood outside of normality, the ordinary, the expected, permeated with fears, insecurities, uncertainties, terror, uneasiness, disturbance and anguish.

*We get COVID and think we’re going to die; we’re going to go down the tube. It was horrible, it was a really bad experience, wow!* [...] *34 weeks of pregnancy* [...] *they started inducing labor, I was in a lot of pain, it wasn’t the way I imagined, the way I wanted* [...] *I was really scared of losing my baby, of staying in the hospital for so long, because no one knew what was going to happen* [...]. (W3)

[...] *I was 7 months pregnant; they were trying to do everything possible to avoid intubation* [...] *they kept me on medication, sedated me, I slept all the time* [...] *I got much worse. They woke me up and told me that I was going to have to have a cesarean section, that my baby was at risk* [...] *I was very dizzy, I had high blood pressure, I had diabetes* [...] *they said it could get worse* [...]. (W4)

### Postpartum and breastfeeding experiences and pandemic limitations

The postpartum period for some women was experienced in rooming-in with the baby. For other women, due to the preterm birth, mother and NB were separated for different reasons, which included the need for intensive care by the mother or baby, or the baby being discharged home and the mother remaining in the Adult ICU.

In shared accommodation, restrictions on access for companions intensified loneliness and fear. The difficulties were greater with regard to childcare, breastfeeding and postpartum care, especially for those undergoing cesarean section. Healthcare professionals assisted with breastfeeding, which was compromised for most women.

[...] *I was scared to death there alone, I had to turn around, get up from that high bed alone to go take a shower* [...] *I planned to have a normal birth* [...] *come home and take care of him normally like I took care of the others and without feeling that pain* [...] *until now, I still feel pain* [cesarean] [...] *I was very sad because I would really like my husband to be there, he wanted to participate* [...] *it’s really bad to feel alone* [...]. (W2)

[...] *I went to the room, and then I couldn’t be near her* [...] *I only got close to her when it was time to breastfeed. The nurse came, put her to breastfeed, she breastfed and the nurse took her away and took her to the crib* [...] *she stayed away from me* [...] *she needed a milk donation. Because of COVID, my milk took a while to come in* [...]. (W1)

[...] *post-cesarean, having to take care of the baby alone, it was really bad* [...] *I imagined that I would have someone to stay with me* [...] *my fear was being alone and not being able to have anyone with me.* [...] *he* [professional] *always asked if he* [the newborn] *was able to breastfeed, you know, if he was latching on well, the nurses came, helped me, gave me guidance* [...]. (W9)

Breastfeeding was also compromised for women and NBs who were in the ICU, although some had support and encouragement from nursing professionals, including from the milk bank. However, it is worth noting that, with great effort, some women managed to breastfeed and maintain exclusive breastfeeding.

[...] *I couldn’t breastfeed my son* [...] *the baby has to have an ideal weight to breastfeed, one kilo and seven hundred* [...] *he reached one kilo and seven hundred, but the milk didn’t come out as much anymore, very little milk came out,* [...] *despite me going to the milk bank every day* [...] *trying to stimulate him to see if the milk wouldn’t dry up* [...]. (W15)

[...] *one thing I really wanted was to be able to breastfeed. They took it away for me at the hospital because I didn’t have the strength* [...] *I couldn’t hold my arm up, with all the equipment* [...]. *When I came home, I did the stimulation, I took it out with the machine at home and it went on, I was able to breastfeed him* [...] *at first, I had to supplement, because my milk still came down little* [...] *when I came home with him, when he was discharged, my milk came down like this, completely* [...] *to this day he only breastfeeds.* (W8)

The speeches of women whose children were hospitalized in intensive care reflect the fragility and finitude of human beings, intensified by the pandemic, including the little being that has just been born. Worry, anguish, fear and uncertainty were part of these women’s daily lives for days on end. Seeing their child for seconds after birth, and not being able to touch or hold them made the experience difficult. There was an uncertain possibility of the NB’s recovery. An unpredictable future amid the complexity and chaos of the pandemic.

[...] *it was horrible not being able to even hold your baby, very sad indeed,* [...] *he was intubated, I couldn’t even see my baby’s face* [...] *they were afraid that I would transmit COVID* [...] *he was hospitalized for 38 or 39 days* [...]. (W10)

[...] *I didn’t see my son cry. He came out of my belly, I didn’t even look at his little face, because they didn’t let me* [...] *he was intubated right away* [...] *I stayed in the hospital for about five days, came home, completed quarantine, and after 14 days, I went to see my son for the first time* [...]. *I was very scared* [...] *at 31 weeks, he wasn’t ready* [...] *we don’t know what would really happen* [...] *if COVID would pass to him.* (W15)

Women who needed to be admitted to the Adult ICU (some of whom had their child in the neonatal ICU) described anguish and sadness related to being away from their child and family, in the face of the unexpected, unpredictable and uncertain moment.

[...] *my case was very serious, I was really sick* [...] *I was afraid of dying and not having anyone to leave my son with* [...] *I cried a lot, I couldn’t wait to get out of there* [ICU]. *I was in agony, distressed, because I wanted to see the baby* [...]. (W4)

[...] *I thought I wouldn’t get out of there* [ICU] *alive. I never imagined that I would need to be intubated one day.* [...] *I got to see him 13 days after he was born, it was very sad for me, very difficult* [...] *my biggest fear was losing my son and not being able to take care of him, not being able to see him grow up* [...]. (W8)

### (Lack of) assistance to women in the process of childbirth and postpartum

In the experience of care, for some women, healthcare professionals demonstrated human care and empathy, provided guidance, support, and conveyed calm and tranquility. They were patient and concerned about their well-being. They made the experience positive and respectful, with a sense of care, even with greater work demands due to the pandemic.

[...] *they were all very kind to me, they were like angels in my life, they all* [...] *guided me a lot* [...] *a premature baby, they already knew that I would have more difficulties* [...] *they were always supporting me* [...] *keeping me informed of what would really happen when he was born.* [...] *nurse, resident doctor* [...]. (W3)

*It was a normal birth* [...]. *The doctor did everything he could to make it happen at the right time* [...]. *The pain in a normal birth is horrible, but the doctor was really good* [...]. *And the nurse was a 10* [...]. *The doctor was very professional, he let my baby be born in the way I found most comfortable, on his knee* [...] *even though he was premature* [33 weeks]. *He didn’t need to be in the ICU, he went straight to the room. God sent a blessed nurse* [...] *she gave me the support I needed* [...]. (W5)

[...] *contractions started* [...] *the nurses stayed by my side the whole time providing assistance. That was what helped me the most* [...] *she* [the doctor] *was very kind, very attentive. They provided a lot of assistance* [...] *they were very patient, they talked a lot* [...] *the doctor, the nurses, they were all very patient, they talked a lot, they tried to give us a lot of strength and security.* (W11)

Contrary to what was stated above, the experience of care was also negative. In particular, care during childbirth presented setbacks. It was possible to note situations such as leaving the postpartum women alone, in isolation, without professional assistance, leaving postpartum women without eating or drinking liquids, ignoring childbirth and birth plan and/or desire for natural birth, when there was no implication of risk, and request for the presence of a companion not being accepted.

[...] *I didn’t eat or drink water, because they said they might have to do a cesarean* [...]. (W14)

[...] *Even though there were a lot of people there, there was no nurse there by my side when I was there feeling those cramps, that horrible pain. I had to feel that pain alone* [...] *there was no one there to help me.* (W2)

*I was hospitalized for a week, taking medicine to hold my baby* [...]. *They took me to the obstetrics center, put me on a stretcher in the office and locked me in there, no one gave me any support there, they left me there.* (W5)

[...] *I did the entire childbirth plan* [...] *I have a childbirth plan authorizing me to induce my dilation* [...] *I went to the surgery alone, there was no one with me* [...]. (W7)

[...] *I always said that I wanted to have a natural birth* [to the prenatal doctor] [...] *I never imagined that it would be like this* [...] *they said I would have to have a cesarean section* [...]. (W9)

Postpartum care, already experienced in the COVID-19 ward, presented setbacks or was contrary to what is expected of human care, through attitudes of neglect, negligence, incompetence, inattention, and lack of information. It is worth highlighting that, although the professionals in the COVID-19 ward were not experts in maternal and child care, human care is inherent to any healthcare professional, as is the adequate execution of health techniques and procedures.

*My experience was horrible* [...]. *They didn’t even give us water. I asked for water, my mouth was dry, and they said they would bring water and they didn’t*. [...] *a rude nurse came to the room and said* [...] *that they would throw away everything that had come into the room* [...] *my clothes, my baby’s clothes* [...] *that everything would be thrown away because it couldn’t be used* [...]. *They weren’t taking care of me; they weren’t doing their job. When you’re in the hospital, you have to be supported. I wasn’t respected, I was treated very badly* [...] *the nurses themselves said they weren’t prepared to receive a mother and a baby in the COVID ward, because they didn’t know how to care for them. They only took care of adults, of sick, big people, and not of little babies.* [...] *the way they treated me was inhumane* [...] *they denied my son supplements when he needed supplements, my son was starving* [...]. (W5)

[...] *because there* [COVID-19 unit] *was not the ward where these people who have babies stayed* [...] *it seems that they* [nursing professionals] *were a bit lost* [...] *when I asked about the COVID result, which they did on the baby, no one told me, the lady went there, said she would check for me, and never came back, they didn’t give me the right information* [...] *there in the COVID ward they are not used to children, to people who have just had a baby* [...] *someone came and said that they could bring in my clothes and the baby’s, then another one came and said that they couldn’t, you know* [...] a*nother one said that the baby’s father, after the baby was born, would be able to come in, and then he couldn’t come in* [...]. (W9)

Isolation in a COVID-19 unit - in a ward or ICU - intensified loneliness, anguish, discomfort and fear of death. The movement of professionals to care for more serious patients, the distinct noises or sounds (coughing or difficult breathing of other patients, equipment that alarmed and others) worried, frightened and caused fear. A scenario of instability and uncertainty. Regarding protective measures, it was observed that the use of caps, masks, gloves, specific clothing, shoe covers and contact restrictions by professionals was observed. As for isolation, it was noted that some were alone, others shared a room with more people.

*I was admitted to the COVID ward, in a room by myself*, [...] *then I was admitted to the ICU* [...] *a room that had three other old ladies. All three were intubated* [...]. *I was in that corner, alone* [...] *loneliness, fear of not going home.* (W4)

[...] *In the room I was in, there was me and another girl, I barely slept* [...] *I was more terrified* [...] *some elderly people were coughing, almost dying from coughing.* (W2)

[...] *You see, like, people getting worse, people feeling sick all the time, others being discharged and others going to the ICU* [...] *a very bad feeling.* (W15)

[...] *I couldn’t have anyone with me or receive visitors* [...] *I would only take her with a mask, with alcohol and a mask, 24 hours with a mask, until she was 15 days old* [...] *I stayed in a room alone* [...] *she was the first baby to be born in the COVID ward* [...] *on the first night, before she was born, there were people calling the nurses in despair, people trying to breathe, who made a noise that I can’t explain as if they were taking a very strong breath. I was very scared* [...]. (W14)

*It’s not easy being alone. It gives you a feeling of abandonment* [...] *of being abandoned in a place.* (W1)

Communication with family was mediated by professionals, via phone calls or video or voice calls using a mobile phone app. Not all women were allowed to keep their mobile phones with them. Those who were deprived of the use of their mobile phones felt more alone, abandoned, and missed their family.

They found some peace of mind and emotional comfort through contact via cell phone, voice or video calls with their family, and when they could see their child, even via video call, mediated by healthcare professionals, a priori, a social worker and a doctor.

[...] *they made videos of him there* [in NB] *I knew he was well taken care of, that comforted me a little* [...]. (W8)

*By cell phone, video call* [...], *when I didn’t make a video call* [...] t*he COVID doctor, he would take the cell phone, I think it was from the hospital, or his, I don’t know, he would make a video call, explain my condition, show the baby, that the baby was fine.* (W7)

*It was a social worker* [who spoke to the family], *because when I was intubated, they put my cell phone in a bag to give to my mother.* [...] *So, I missed it so much, my heart ached, I felt alone.* (W12)

## DISCUSSION

Because pregnant women have a suppressed immune system, there is a higher risk of developing serious or critical illnesses associated with COVID-19, in particular pneumonia and respiratory failure, leading to hospitalization and early delivery, in addition to a higher risk of premature rupture of membranes, premature delivery, miscarriage, pre-eclampsia and cesarean delivery^(9-11)^. Regarding perinatal outcomes, there is an increased risk of fetal distress, Apgar <7 at the fifth minute, neonatal asphyxia, low birth weight, admission to the neonatal ICU, and perinatal death^(9,10)^.

Given these clinical data and women’s experiences, the pandemic reveals human complexity and fragility amid uncertainties, unknown and unexpected situations related to pregnancy, childbirth and birth. These conditions are linked to existential challenges, which reveal the strengths and weaknesses of human beings, the control and lack of control over life, the uncertain future and the close relationship between human beings and death^(2)^.

Birth is highly anticipated by families, but not preterm birth, which is unexpected and often requires admission to a neonatal ICU. Thus, when a NB is separated from their parents, it affects them emotionally throughout hospitalization, and can trigger negative emotions, especially in mothers^(12)^. This condition is intensified by the restrictions of the pandemic^(3)^ in which, in some cases, neither parent was able to see, touch or be with NBs. These factors aggravated the already high levels of suffering of women^(6,13)^.

A global study of 2,103 participants from 56 countries found that 52% of respondents were not allowed to have anyone else present during the birth. The increase in percentages was related to restrictions in the respondents’ country of residence. Furthermore, 21% of respondents indicated that no one was allowed to be with the baby in the ICU. The frequency and duration of the presence allowed depended on the extent of the restrictions^(14)^.

NB hospitalization in ICUs and restrictions on access by parents due to COVID-19 consequently created obstacles to early bonding between mother/father and child, increasing loneliness and isolation for mothers who were infected. Therefore, the protective measures inherent to the pandemic proved to be unfavorable for parents^(15)^.

Among the adversities, we can point out the impairment of exclusive breastfeeding after childbirth, identified by women’s reports, related to separation of NBs from their mothers and to self-efficacy in breastfeeding. Thus, mothers’ emotional factors, impairment of their bond with NBs and the lack of social support may have interfered with breastfeeding^(16)^.

According to comprehensive studies, a significant proportion of women who experienced childbirth during the COVID-19 pandemic had a negative impact on their perinatal mental health, experiencing depression, sadness, anxiety, insecurity and comorbid symptoms, and the emotional condition of women can harm breastfeeding^(4,6,13)^.

Despite the difficulties experienced, some women managed to continue breastfeeding their children, with the support of nursing professionals from the Human Milk Bank (HMB). This demonstrates the importance of this service for the protection, promotion and support of breastfeeding, particularly for mothers of babies who cannot breastfeed due to prematurity^(17)^.

Donor milk becomes important when the mother of preterm babies has low or no milk production. Increasing levels of health education about the functioning and importance of HMB can have a positive impact on the recruitment of these donors^(18)^.

During childbirth, birth and postpartum, given the need to reduce the risk of viral transmission, women’s rights ceased to be a priority, and current scientific recommendations began to be followed, such as reducing the circulation of people, assessing the need to terminate pregnancy, preferring epidural anesthesia to general anesthesia, encouraging breastfeeding, although skin-to-skin contact was contraindicated and it was recommended to keep women isolated from NBs^(11)^. Some are even contradictory.

According to the paradigm of complexity^(2,7)^, contradictions, order and disorder, balance and imbalance, chaos and adversity are part of everyday experiences. In the midst of this, during the pandemic, healthcare professionals continued their activities, exposed to contagion and death, as missionaries. They maintained interactions and interrelations for obstetric care during childbirth and postpartum, adapting to the ways of caring. Among the professionals, some demonstrated essentially human and supportive attitudes, but others practiced acts contrary to the expected human care.

These situations, although experienced in a chaotic scenario due to the pandemic, are linked to human action, which demonstrated disregard for others - the woman and the NB. A movement that contradicts human care, a complex moment not understood by all professionals who provide care, with recommendations for childbirth care that were not followed.

Studies confirm antagonisms in care. On the one hand, women were satisfied with the care provided by professionals. These professionals, through their actions and words, promoted comfort, tranquility, pain relief, well-being, respect for autonomy and appreciation of women during the birth process^(19)^. They practiced sensitive and empathetic care, amidst increased work demands and fear of viral contamination^(4)^.

On the other hand, women experienced feelings of abandonment, discomfort, neglect of care, with verticalized relationships between women and professionals^(19)^, particularly when choosing the route of childbirth^(4)^. In view of this, unnecessary exposure to women and NBs was added, with risks of unexpected outcomes^(19)^, intensified by COVID-19 infection^(9)^.

These conditions represent setbacks in childbirth care. Regarding the work of professionals involved in the parturition process, the importance of meeting women’s human-affective needs is highlighted, in addition to the technical needs related to childbirth and birth. It is stated that women need to be considered as a single and multiple being, with their weaknesses, fears and uncertainties inherent to the moment experienced. Leaving a postpartum women alone or deciding to be with her implies an act of solidarity and humanity inherent to human beings^(2,7)^, an attitude expected of healthcare professionals responsible for care.

In Brazil, the right to have a companion present during childbirth has been supported by Federal Law 11.108 since 2005^(20)^. Although it was recommended that women be monitored during the pandemic^(11)^, their exclusion was intended to promote safety for women, babies, and healthcare professionals^(3)^. This condition violates this right and has had an emotional impact on women. It is worth noting that, in 2023, Law 14.737 was published, which guarantees women monitoring by a healthcare professional indicated by the institution; if the woman does not indicate a companion, in the case of sedation or reduced level of consciousness or in the surgical center^(21)^.

The childbirth and birth plan, which expresses women’s preferences, desires and expectations regarding childbirth/birth^(22)^, is indisputably relevant to the effectiveness of human dignity, the right to health and the physical and psychological integrity of women in the labor process^(23)^. It is also positive for scheduled cesarean sections, given that all women, regardless of the route of delivery, can receive respectful care, as they wish^(24)^. Therefore, ignoring the childbirth and birth plan or women’s wishes implies carrying out acts against their will^(23)^. However, most women are unaware of the childbirth and birth plan^(25)^ and do not exercise their autonomy.

It should be added that women affected by COVID-19 who wanted natural birth could have it, as long as clinical and obstetric assessments were favorable, with surgical termination of pregnancy being possible when assessments were unfavorable and/or there were changes in fetal vitality^(11)^. Thus, even if women’s wishes are not expressed in a document, it is necessary to respect what she wants^(24)^, considering the absence of clinical and/or obstetric risks during childbirth and birth^(11)^.

However, women’s autonomy depends on cultural and social conditions. Women are capable of making choices, taking decisions, and examining their behaviors with autonomy and freedom, but they are also subject to heteronomy, since the fulfillment of their choices depends on the professionals who assist them and other conditions over which they have no control. In this logic, women may have the impression that they are free in their choices, without being^(7)^.

Isolation in an environment restricted to people with COVID-19 (ward or ICU), without a companion, with the need to wear a mask (although some refused to wear one), caused suffering to women^(4,26)^. The fact that they were assisted by professionals who were not always those with training or qualifications in obstetrics created a barrier and compromised care.

Due to the absence of a companion during labor and isolation, healthcare professionals used strategies to bring women closer to their families through video calls on their cell phones to provide emotional support to women^(4)^. The need for greater support for those who presented suffering and consequent impairment of perinatal mental health is highlighted^(6,13)^.

Although psychological care for women who experienced childbirth, birth and postpartum during COVID-19 was recommended to minimize the suffering experienced^(26)^, this was not confirmed by statements. Nursing and medical professionals were the ones who mainly sought to alleviate the challenges of women’s experiences, offering emotional support, followed by social workers, who mediated contact with the family.

### Study limitations

The study had the limitation of having conducted interviews with women who experienced childbirth and birth in a single hospital, which was nevertheless a reference for childbirth of women with COVID-19.

### Contributions to nursing, health or public policy

The study contributes to reflections on maternal care, in light of complexity, in unexpected contexts of uncertainty and contradictions, such as the COVID-19 pandemic.

## FINAL CONSIDERATIONS

It was understood that the experience of childbirth/birth/postpartum was different from what women expected, permeated by unpredictability and uncertainty, intense emotions and meanings that caused suffering to women. The restrictions on contact and the absence of a companion compromised their mental health, NB care and self-care in the postpartum period. The bond with NB and breastfeeding were compromised.

Some peace of mind and emotional comfort could be experienced by women when they were able to have contact with their family or see the preterm NB (who was in the neonatal ICU), through voice or video calls, commonly mediated by healthcare professionals.

As for the experience of care, antagonistic conditions were noted. While some healthcare professionals demonstrated attitudes of humane care, others did not. Setbacks in practices consistent with obstetric care were identified.

It is recommended that women’s rights already achieved in childbirth care, guaranteed by law, be respected, such as the presence of a companion, as well as compliance with current obstetric recommendations in childbirth and birth care, designation of trained professionals imbued with care to assist women and NBs in rooming-in and monitoring of women who experienced childbirth during COVID-19 diagnosis, in terms of mental health, by Primary Healthcare professionals.
