# Supplementary material for: Uncertainties and contradictions experienced by women with COVID-19 during childbirth/birth and the postpartum period
Source: Rev Bras Enferm. 2025 Mar 14;78(1):e20240236. doi: 10.1590/0034-7167-2024-0236 (PMC11913041; doi:10.1590/0034-7167-2024-0236)
Supplement: Translation

# Translation: Incertezas e contradições experienciadas por mulheres com COVID-19 no parto/nascimento e período pós-parto

**DOI:** 10.1590/0034-7167-2024-0236pt

**Authors:** Maria Aparecida Baggio, Amanda Martins de Souza, Rosane Meire Munhak da Silva, Marli Terezinha Stein Backes, Adriana Zilly

**Affiliations:** I Universidade Estadual do Oeste do Paraná. Cascavel, Paraná, Brasil; II Universidade Estadual do Oeste do Paraná. Foz do Iguaçu, Paraná, Brasil; III Universidade Federal de Santa Catarina. Florianópolis, Santa Catarina, Brasil

**Keywords:** Mulheres, Parto, Período Pós-Parto, COVID-19, Obstetrícia

## Abstract

**Objetivos:**

compreender as experiências e os significados atribuídos ao parto/nascimento e pós-parto por mulheres acometidas por COVID-19.

**Métodos:**

estudo qualitativo à luz da complexidade, conduzido por entrevistas com 15 mulheres em hospital universitário referência para gestantes infectadas e de alto risco da décima Regional de Saúde do Paraná, Brasil, entre dezembro/2021 e abril/2022. Dados foram analisados pela análise temática.

**Resultados:**

identificaram-se experiências de parto/nascimento e pós-parto diferentes do desejado e planejado, permeadas de incertezas, imprevisibilidades e contradições, sobretudo quanto aos desfechos maternos e neonatais. Experiências repletas de significados e emoções causaram sofrimento e comprometimento da saúde mental, particularmente relacionadas à prematuridade, afastamento do recém-nascido, ausência do acompanhante e isolamento em unidade COVID-19. Constataram-se prejuízos no vínculo mãe-bebê, autocuidado e amamentação, e atuações profissionais antagônicas, com retrocesso às práticas obstétricas.

**Considerações Finais:**

aponta-se a necessidade de respeito aos direitos assegurados por lei e designação de profissionais capacitados para a assistência materno-infantil.

## INTRODUÇÃO

A pandemia de COVID-19 foi declarada pela Organização Mundial da Saúde (OMS) em 11 de março de 2020^(1)^, paralisando a vida econômica e social no mundo e dando origem a uma catástrofe sanitária de saldo de mortes alarmantes. Criou-se uma crise, impelindo ao ser humano múltiplas reflexões e adequações dos hábitos de vida e de trabalho à condição pronunciada. Seres humanos ficaram confinados em minúsculos espaços (seja para se proteger ou se tratar, quando vitimados) e abertos a um incerto destino^(2)^.

Na área da saúde, para o enfrentamento da pandemia, nas suas diferentes fases, foi necessária a adoção de estratégias de gestão do cuidado de pessoas infectadas pelo vírus ou não, por gestores e profissionais dos serviços, globalmente. Para isso, foi reconfigurada a dinâmica de atendimento em hospitais, com adequação de estrutura física e de processos de trabalho, gerenciamento de recursos humanos, implementação de estratégias de prevenção de infecção pelo vírus SARS-CoV-2 e garantia de segurança de profissionais e usuários, de acordo com as recomendações nacionais, regionais e internacionais. Em especial, hospitais universitários foram requisitados para oferta de leitos de enfermarias e Unidade de Terapia Intensiva (UTI) para portadores da infecção^(3)^.

Por conta da pandemia, o atendimento obstétrico em hospitais também passou por adequações. Novas demandas surgiram e, consequentemente, novos modos de assistência foram incorporados aos serviços, concomitante ao retrocesso de outros. O desafiante controle da pandemia, por medidas protetivas, aumentou os desafios dos profissionais de saúde para a assistência ao parto^(4)^ e pós-parto.

A assistência nesses períodos de transição requer apoio intensivo às mulheres pelos profissionais de saúde. Contudo, mudanças na experiência do nascimento, preocupações sobre a transmissão do vírus, atraso no cuidado, separação de recém-nascidos (RN) de suas mães, restrições de acompanhantes e dificuldade em estabelecer contato com eles foram algumas das condições comuns em maternidades que fragilizaram o cuidado^(5)^.

Estudo realizado nos Estados Unidos acerca da experiência de mulheres durante a gravidez, parto/nascimento e pós-parto, na vigência da pandemia de COVID-19, pontuou que as experiências variaram, com base na localização geográfica, paridade e suporte que elas possuíam, sobretudo o emocional. A pandemia afetou as mulheres e suas famílias, principalmente quanto às expectativas *versus* realidade das experiências que tiveram^(6)^.

Diante do exposto, questionou-se: quais foram as experiências e os significados atribuídos ao parto/nascimento e pós-parto por mulheres acometidas por COVID-19?

## OBJETIVOS

Compreender as experiências e os significados atribuídos ao parto/nascimento e pós-parto por mulheres acometidas por COVID-19.

## MÉTODOS

### Aspectos éticos

O estudo é parte de projeto maior intitulado “Enfrentamento da covid-19 e a assistência materno infantil”, que atendeu à Resolução nº 466/2012 do Conselho Nacional de Saúde e Pesquisa Envolvendo Seres Humanos. Para garantir o anonimato, as participantes foram identificadas pela letra “M”, relativa à mulher, seguida por numeração arábica, conforme a ordem da entrevista (M1... M15).

### Referencial teórico-metodológico

A teoria da complexidade foi o referencial teórico utilizado^(2,7)^.

### Tipo de estudo

Estudo qualitativo, com utilização do *COnsolidated criteria for REporting Qualitative research* (COREQ) como guia para orientar o relatório do processo da pesquisa.

### Cenário do estudo

Hospital universitário de Cascavel, Paraná, Brasil, referência para gestantes infectadas e de alto risco, da décima Regional de Saúde do Paraná.

### Procedimentos metodológicos

Participaram do estudo 15 mulheres que tiveram parto na vigência do diagnóstico de COVID-19. A seleção das participantes aconteceu a partir de uma lista de 97 gestantes admitidas no hospital do estudo, com infecção por COVID-19, entre março de 2020 e março de 2022, informada pelo serviço de vigilância epidemiológica. A seleção das mulheres foi por conveniência, mediante critérios de inclusão. Foi realizado contato telefônico com 47 mulheres, das quais 15 aceitaram participar. Além disso, 22 não aceitaram; com cinco não foi possível contato (número de telefone incorreto); as famílias de três mulheres informaram ocorrência de óbito pós-parto; e duas não falavam o idioma português.

Foram incluídas mulheres com mais de 18 anos, que tiveram parto na vigência do pico da infecção por SARS-CoV-2 no hospital público, referência para internação de gestantes com suspeita ou diagnóstico de COVID-19, independentemente da idade gestacional. Mulheres que não falavam a língua portuguesa, com problemas de saúde física ou mental que impedissem sua participação e aquelas que evoluíram ao óbito foram excluídas.

### Coleta e organização dos dados

Mediante o aceite de participar na pesquisa, foram marcados dia e horário para a entrevista, conforme disponibilidade da participante. Foram realizadas 12 entrevistas por meio de ligação de voz e duas por ligação de vídeo, pelo aplicativo *WhatsApp^®^,* e uma presencialmente, no local do estudo, com duração média de 30 minutos, com garantia de privacidade. Foram realizadas três entrevistas piloto, que foram incluídas no estudo por alcançarem profundidade à temática em pauta. Não houve necessidade de ajuste do instrumento.

As entrevistas foram realizadas por uma graduanda em enfermagem, treinada e acompanhada pela pesquisadora responsável, que possui *expertise* em pesquisa qualitativa. O período de coleta de dados foi entre dezembro de 2021 e abril de 2022, por meio de entrevista individual, guiada por um roteiro semiestruturado, iniciado pelas perguntas disparadoras: fale-me sobre sua experiência de parto e pós-parto. Comente sobre a experiência do nascimento do(a) seu(a) filho(a). Fale sobre os significados da sua experiência.

As entrevistas foram audiogravadas, transcritas e devolvidas às participantes pelo *WhatsApp^®^* para correções do conteúdo transcrito. Nenhuma participante acenou correção. O fenômeno da saturação permitiu encerrar as entrevistas.

### Análise dos dados

Os dados foram analisados por meio da análise temática^(8)^, composta por três etapas. Na primeira etapa, pré-análise, foi realizada a leitura dos dados para atingir exaustividade, representação, homogeneidade e pertinência, a partir do objetivo proposto. Na segunda etapa, exploração do material, foi realizada a classificação dos dados, considerando-se as expressões e/ou palavras significativas, de acordo com seus núcleos de sentido, para identificar categorias e subcategorias. Na terceira etapa, tratamento dos dados e interpretação, as inferências e interpretação do conteúdo de análise foram fundamentadas pelo referencial teórico adotado neste estudo^(2,7)^.

## RESULTADOS

As mulheres participantes acessaram o hospital por sinais de trabalho de parto, por suspeita ou diagnóstico de COVID-19 e/ou por complicações respiratórias. Por ser referência em alto risco, o hospital admitiu mulheres para o parto e/ou para tratamento de complicações da COVID-19 também de outro município, de outra regional de saúde do Paraná, como do município Assis Chateaubriand, da vigésima Regional de Saúde de Toledo.

A maioria delas era sintomática, com gestação pré-termo, multípara, média de idade de 32,9 anos, e duas tiveram óbito perinatal como desfecho. Das dez mulheres em que a via de nascimento foi cirúrgica, quatro manifestaram o desejo de parto vaginal, uma delas com plano de parto e nascimento. Algumas disseram terem sido infectadas pelo companheiro, mas a maioria não sabia informar o contato. Vacinas ainda não estavam disponíveis às gestantes na época do estudo (Quadro 1).

**Quadro 1:** Caracterização das mulheres em relação a dados maternos, obstétricos, da COVID-19 e procedência, Cascavel, Paraná, Brasil, 2021

M	Idade	IG	Paridade na admissão	Motivo da admissão	Sintomas na admissão	Via de nascimento	Procedência	Período: parto e entrevista
M1	42	38	GIVAICII	TP	Sim	Cirúrgica	Cascavel	7 meses e 5 dias
M2	35	37	GIVPIII	BR	Sim	Cirúrgica	Catanduvas	6 meses e 22 dias
M3	25	34	GIIIPII	BR	Sim	Vaginal	Cafelândia	9 meses e 14 dias
M4	31	31	GIIAI	Complicação	Sim	Cirúrgica	Assis Chateaubriand	1 ano, 5 meses e 24 dias
M5	32	34	GI	TP	Sim	Vaginal	Cascavel	10 meses e 23 dias
M6	26	28	GI	TP	Não	Vaginal^*^	Cascavel	1 ano, 3 meses e 17 dias
M7	36	40	GI	TP	Sim§	Cirúrgica#	Cascavel	10 meses e 23 dias
M8	26	32	GI	Complicação	Sim§	Cirúrgica	Capitão Leônidas Marques	1 ano, 1 mês e 3 dias
M9	27	38	GIICI	BR	Sim	Cirúrgica	Cascavel	8 meses e 2 dias
M10	43	31	GIVAIPII	Sintomas	Sim§	Cirúrgica	Cascavel	1 ano, 1 mês e 22 dias
M11	39	38	GIIAI	Sintomas	Sim§	Vaginal^*^	Cascavel	1 ano, 10 meses, 10 dias
M12	41	32	GVIIAIIPIV	Complicação	Sim	Cirúrgica	Cascavel	11 meses e 15 dias
M13	27	32	GIIICII	Complicação	Sim	Cirúrgica^*^	Cascavel	8 meses e 11 dias
M14	22	39	GI	TP	Não	Vaginal	Quedas do Iguaçu	1 ano, 3 meses e 20 dias
M15	42	31	GIVAIPII	Sintomas	Sim§	Cirúrgica	Cascavel	1 ano, 2 meses e 23 dias

* *Óbito perinatal; #Plano de parto e nascimento; §Companheiro positivo; M - mulher; IG - idade gestacional; G - gestação; P - parto normal/vaginal; A - aborto; C - cesárea; TP - trabalho de parto; BR - bolsa rota.*

Os aspectos relacionados aos significados do parto e nascimento, experiências no pós-parto e amamentação e (des)assistência à saúde permitiram organizar três categorias de análise.

### Significando a experiência do parto e nascimento

Foi possível identificar, nas experiências das mulheres, o inesperado, o imprevisível e o incerto relacionado ao parto e nascimento. O inesperado esteve atrelado a múltiplas condições, quais sejam: ao trabalho de parto prematuro, desencadeado pela COVID-19; ao trabalho de parto, mesmo que a termo, mas na vigência da pandemia de COVID-19; às complicações clínico-obstétricas decorrentes da infecção pelo SARS-CoV-2; à experiência do parto não planejado (cirúrgico ou induzido); ao local de parto (centro cirúrgico ou unidade COVID-19); ao desfecho do nascimento; entre outras condições.

Algumas mulheres tiveram a gestação interrompida por indução de parto ou por cirurgia cesariana. Pelos depoimentos, o desejo da maioria era o parto vaginal, embora não tivessem um plano de parto e nascimento. Contudo, a maioria foi submetida à cirurgia, em centro cirúrgico, descrito como improvisado, com presença de vários profissionais, alguns demonstrando ansiedade. Duas experienciaram o parto na unidade COVID-19 e, duas, na triagem do pronto-socorro. Foi diferente do imaginado, do planejado, do que elas desejavam.

[...] *eu estava tão apavorada* [...] *para mim, eu ia lá* [centro cirúrgico], *eles iam tirar meu filho* [cesárea] *e eu ia morrer* [...] *estava cheio de enfermeiros, tinha umas quatro, cinco enfermeiras* [...] *médico, todo apavorado, todo ansioso* [...], *eu pensei: “Ele vai me matar aqui!”* [...] *era uma sala improvisada* [...] *tinha medo de perder ele* [bebê] *enquanto eu não vi ele nascer* [...] *que ele estava tudo bem não fiquei sossegada.* (M2)

*Tinha as enfermeiras da ala do COVID e vinha o obstetra* [...] *ela nasceu* [na ala COVID-19] [...] *tinha as enfermeiras ali junto e, na hora que ela foi nascer, veio a obstetra. Quando ela entrou no quarto, que ela foi fazer o toque e daí estourou a bolsa* [...] *ela falou que ela ia nascer ali naquela sala mesmo* [...]. (M14)

*Eu estava dentro da ambulância, fiquei uma hora até eles* [equipe do hospital] *achar uma maca* [...] *eu tive um sangramento* [...] *começou a dar mais dores, contração* [...] *eu tive o bebê ali embaixo mesmo* [triagem] *prematuro, com 7 meses* [...]. *Ela* [médica] *pegou o bebê e saiu correndo com ele* [...] *ela foi lá para o neonatal* [UTI] *para tentar intubar o bebê* [...] *eu não sei o que aconteceu lá em cima* [...] [RN foi a óbito]. (M6)

O imprevisível e o incerto estiveram relacionados ao estado clínico-obstétrico das mulheres e às respostas maternas e neonatais após o parto e nascimento. Viver ou morrer era incerto - para mãe e filho. A finitude do ser humano estava em pauta, aflorando um universo de significados. Uma experiência de maternidade fora da normalidade, do comum, do esperado, permeada de medos, inseguranças, incertezas, pavor, intranquilidade, perturbação e angústia.

*A gente pega COVID e acha que vai morrer, vai para o tubo. Foi horrível, foi uma experiência bem ruim, nossa!* [...] *34 semanas de gestação* [...] *começou a induzir o parto, eu tive muita dor, não foi do jeito que eu imaginava, do jeito que eu queria* [...] *eu tive muito medo de perder meu bebê, de ficar tanto tempo no hospital, porque ninguém sabia o que ia acontecer* [...]. (M3)

[...] *eu estava gestante de 7 meses; eles estavam tentando fazer o impossível para não entubar* [...] *me mantendo com medicação, sedada, eu dormia o tempo todo* [...] *piorei muito. Eles me acordaram e falaram para mim que eu ia ter que fazer cesárea, que o meu bebê corria risco* [...] *eu estava bem atordoada, eu tinha pressão alta, eu tinha diabetes* [...] *eles falaram que poderia agravar* [...]. (M4)

### Experiências do pós-parto e amamentação e as limitações da pandemia

O período pós-parto de algumas mulheres foi experienciado em alojamento conjunto junto ao bebê. Para outras mulheres, visto o nascimento pré-termo, mãe e RN foram afastados, por situações distintas, que incluíram a necessidade da mãe ou do bebê de cuidados intensivos, ou alta do bebê para casa e mãe permanecendo na UTI Adulto.

No alojamento conjunto, as restrições de acesso para acompanhantes intensificaram a solidão e o medo. As dificuldades foram maiores no que concerne aos cuidados do filho, amamentação e cuidados próprios do puerpério, principalmente às submetidas à cesariana. Os profissionais de saúde auxiliavam a amamentação, que se mostrou comprometida para a maioria das mulheres.

[...] *fiquei morrendo de medo lá sozinha, tive que me virar, levantar daquela cama alta sozinha para ir tomar banho* [...] *eu planejava parto normal* [...] *vir para casa cuidar dele normal igual cuidei dos outros e sem sentir aquela dor* [...] *até agora, ainda sinto dor* [cesárea] [...] *fiquei muito triste porque eu gostaria muito que o meu esposo estivesse lá, ele queria participar* [...] *é muito ruim a gente se sentir sozinha* [...]. (M2)

[...] *eu fui para o quarto, aí eu não pude ficar perto dela* [...] *só me aproximava na hora de dar de mamá. A enfermeira vinha, colocava ela para mamar, ela mamava e a enfermeira tirava e levava para o bercinho* [...] *ela ficava longe de mim* [...] *ela precisou de doação de leite. Por causa do COVID, meu leite demorou para descer* [...]. (M1)

[...] *pós-cesárea, tendo que cuidar do bebê sozinha, foi bem ruim* [...] *imaginava que eu ia ter alguém para ficar comigo* [...] *o meu medo era ficar sozinha e não poder ficar ninguém comigo.* [...] *ele* [profissional] *sempre perguntava se ele* [RN] *estava conseguindo mamar, sabe, se estava pegando bem, as enfermeiras vinham, me ajudavam, orientaram* [...]. (M9)

A amamentação também se mostrou comprometida para mulheres e RN que ficaram em UTI, embora algumas tivessem apoio e estímulo dos profissionais de enfermagem, inclusive do banco de leite. Contudo, cabe salientar que, com muito esforço, algumas mulheres conseguiram amamentar e manter a amamentação exclusiva.

[...] *eu não pude amamentar meu filho* [...] *o bebezinho tem que ter um peso ideal para mamar, um quilo e setecentos* [...] *ele alcançou um quilo e setecentos, só que já não saía tanto leite, saía muuuito pouquinho leite,* [...] *apesar de eu ir todos os dias no banco de leite* [...] *tentando estimular para ver se não secava o leite* [...]. (M15)

[...] *uma coisa que eu queria muito era poder amamentar. Elas tiravam para mim no hospital porque eu não tinha força* [...] *eu não conseguia ficar com o braço erguido, com todos os aparelhos* [...]*. Quando eu vim para casa, eu fiz a estimulação, eu tirava com a maquininha em casa e foi indo, eu pude amamentar ele* [...] *no início, eu tinha que complementar, porque ainda descia pouco leite* [...] *quando eu vim para casa com ele, que ele ganhou alta, meu leite desceu assim, totalmente* [...] *mama até hoje só o peito.* (M8)

Os discursos das mulheres cujos filhos ficaram internados sob cuidados intensivos refletem a fragilidade e a finitude do ser humano, intensificadas pela condição da pandemia, incluindo o pequeno ser que acabou de nascer. Preocupação, angústia, medo e incerteza fizeram parte do cotidiano dessas mulheres, por dias a fio. Ver o filho por segundos, após o nascimento, não poder tocar ou pegar no colo tornou a experiência difícil. Uma possibilidade incerta de o RN se sair bem. Um futuro imprevisível em meio à complexidade e ao caos da pandemia.

[...] *foi horrível não poder nem pegar seu bebezinho, muito triste mesmo,* [...] *ele foi intubado, não pude ver nem o rostinho do meu bebê* [...] *eles ficaram com medo de eu transmitir o COVID* [...] *ele ficou 38 ou 39 dias internado* [...]. (M10)

[...] *não vi meu filho chorar. Saiu da barriga, eu não olhei nem no rostinho dele, porque não deixaram* [...] *ele logo foi intubado* [...] *permaneci uns cinco dias internada, vim para casa, cumpri quarentena, e depois de 14 dias, fui ver meu filho pela primeira vez* [...]. *Eu fiquei com muito medo* [...] *com 31 semanas, ele não estava preparado* [...] *a gente não sabe o que realmente iria acontecer* [...] *se o COVID iria passar para ele.* (M15)

As mulheres que precisaram ficar internadas em UTI Adulto (de algumas delas o filho ficou na UTI neonatal) descreveram angústia e tristeza relacionadas ao afastamento do filho e da família, diante do inesperado, imprevisível e momento incerto.

[...] *meu caso era muito grave, eu estava bem ruim* [...] *medo eu tinha de morrer e não ter com quem deixar o meu filho* [...] *eu chorava demais, eu não via a hora de sair dali* [UTI]. *Estava agoniada, angustiada, porque eu queria ver o neném* [...]. (M4)

[...] *eu achava que não ia sair viva de lá* [UTI]. *Nunca imaginei que ia precisar algum dia ficar intubada.* [...] *eu consegui ver ele 13 dias depois que nasceu, foi muito triste para mim, muito difícil* [...] *o meu maior medo mesmo era eu perder o meu filho e não poder cuidar dele, não poder ver ele crescer* [...]. (M8)

### (Des)assistência à mulher no processo de parto e pós-parto

Na experiência de assistência, para algumas mulheres, os profissionais de saúde demonstraram cuidado humano e empatia, orientaram, apoiaram, transmitiram calma e tranquilidade. Tiveram paciência e preocupação com o bem-estar delas. Tornaram a experiência positiva e respeitosa, com significado de cuidado, mesmo com maiores demandas de trabalho diante da pandemia.

[...] *todos eles foram muito queridos comigo, foram uns anjos assim, na minha vida, todos* [...] *me orientaram bastante* [...] *um bebê prematuro, já sabiam que eu ia ter mais dificuldade* [...] *elas sempre estavam me apoiando* [...] *me deixando a par do que realmente ia acontecer quando ele nascesse.* [...] *enfermeira, doutora residente* [...]. (M3)

*Foi parto normal* [...]. *O médico fez tudo para na hora ser bom* [...]. *O parto normal, a dor é horrível, mas o médico foi muito bom mesmo* [...]*. E a enfermeira foi nota 10* [...]. *O médico foi muito profissional, ele deixou o meu bebê nascer da forma que eu achei mais confortável, foi de joelho* [...] *mesmo sendo prematuro* [33 semanas]. *Ele não precisou de UTI, foi direto para o quarto. Deus que mandou uma enfermeira abençoada* [...] *ela me deu o suporte necessário* [...]. (M5)

[...] *começaram as contrações* [...] *enfermeiras ficaram o tempo todo dando assistência do meu lado. Foi o que me ajudou mais* [...] *ela* [médica] *foi muito querida, muito atenciosa. Eles deram bastante assistência* [...] *tinham bastante paciência, conversaram bastante* [...] *a doutora, as enfermeiras, todas elas foram bastante pacientes, conversaram bastante, tentavam passar bastante força e segurança para a gente.* (M11)

De modo contrário ao exposto, a experiência de assistência também se revelou negativa. Em particular, a assistência ao parto apresentou retrocessos. Foi possível notar situações como deixar a parturiente sozinha, em isolamento, sem assistência profissional, deixar a parturiente sem se alimentar ou ingerir líquidos, ignorar plano de parto e nascimento e/ou desejo de parto vaginal, quando não havia implicação de risco, pedido de presença de acompanhante não aceito.

[...] *eu fiquei sem comer e sem tomar água, porque eles falaram que talvez teriam que fazer a cesárea* [...]. (M14)

[...] *apesar de ter um monte de gente lá, não tinha nenhuma enfermeira ali do meu lado quando eu estava lá sentindo aquelas cólicas, aquela dor horrível. Eu tive que sentir aquela dor sozinha* [...] *não tinha ninguém lá para me ajudar.* (M2)

*Eu fiquei internada uma semana, tomando remédio para segurar o meu bebê* [...]. *Me levaram para o centro obstétrico, me colocaram numa maca no consultório e me fecharam lá, ninguém me deu suporte nenhum lá, me deixaram lá dentro.* (M5)

[...] *eu fiz todo o plano de parto* [...] *eu tenho um plano de parto autorizando a induzir minha dilatação* [...] *eu fui sozinha na cirurgia, não tinha ninguém comigo* [...]. (M7)

[...] *eu sempre falava que queria ter o parto normal* [ao prenatalista] [...] *nunca imaginei que ia ser dessa forma que aconteceu* [...] *falaram que tinha que fazer cesárea* [...]. (M9)

A assistência no pós-parto, experienciada já na ala COVID-19, apresentou retrocessos ou foi contrária ao que se espera do cuidado humano, por meio de atitudes de descaso, negligência, imperícia, desatenção, falta de informação. Convém grifar que, embora os profissionais da enfermaria COVID-19 não fossem *experts* em assistência materno-infantil, o cuidado humano é inerente a qualquer profissional de saúde, bem como a execução adequada de técnicas e procedimentos em saúde.

*A minha experiência foi horrível* [...]. *Eles não davam nem água para a gente, eu pedia água, eu estava com a boca seca, e eles falavam que iam trazer água e não traziam* [...] *uma enfermeira grossa foi lá no quarto falar* [...] *que tudo o que tinha entrado ali no quarto, eles iriam jogar fora* [...] *minhas roupas, as roupas do meu bebê* [...] *que tudo seria jogado fora porque não poderia ser usado* [...]. *Eles não estavam cuidando, não estavam fazendo o trabalho delas. Quando está no hospital, você tem que ser amparada, eu não fui respeitada, fui muito maltratada* [...] *as próprias enfermeiras falavam que não estavam preparadas para receber uma mãe e um bebê na ala do COVID, porque elas não sabiam como cuidar, elas cuidavam só de adulto, de gente doente, grande e não de bebezinhos.* [...] *a forma que eles me trataram foi desumano* [...] *eles negarem o complemento para o meu filho, sendo que ele precisava de complemento, meu filho passava fome* [...]. (M5)

[...] *porque lá* [unidade COVID-19] *não era ala onde ficavam essas pessoas que ganham nenê* [...] *parece que elas* [profissionais de enfermagem] *estavam meio perdidas* [...] *na hora que eu perguntei sobre o resultado do COVID, que eles fizeram no neném, ninguém me informou, a moça foi lá, falou que ia ver para mim, e não voltou mais, não informavam direito* [...] *lá na ala do COVID eles não estão acostumados com criança, com pessoa que acabou de ganhar neném* [...] *alguém vinha e falava que ia poder entrar a minhas roupas e a do nenê, aí vinha outra e falava que não podia sabe* [...] *uma outra que falou que o pai do nenê, depois que nascesse, ia poder entrar, e daí já não podia entrar* [...]. (M9)

O isolamento em unidade COVID-19 - em enfermaria ou em ambiente intensivo - intensificou a solidão, a angústia, o desconforto e o medo da morte. A movimentação dos profissionais para atender pacientes mais graves, os ruídos ou barulhos distintos (tosse ou respiração difícil de outros pacientes, equipamentos que alarmavam e outros) preocupava, assustava, causava pavor. Um cenário de instabilidade e de incertezas. Quanto às medidas protetivas, constatou-se uso de toucas, máscaras, luvas, roupas específicas, propés e restrição de contato pelos profissionais. Quanto ao isolamento, notou-se que algumas estavam sozinhas, outras dividiam quarto com mais pessoas.

*Fiquei internada na ala COVID, num quarto sozinha,* [...] *depois eu fiquei internada na UTI* [...] *um quarto que tinha mais umas três senhorinhas. As três estavam entubadas* [...]. *Ficava naquele canto, sozinha* [...] *solidão, medo de não voltar para casa.* (M4)

[...] *no quarto que eu estava, estava eu e uma outra moça, quase não dormia* [...] *ficava mais apavorada* [...] *umas pessoas idosas tossiam, de quase morrer tossindo.* (M2)

[...] *você vê, assim, as pessoas piorando, pessoas passando mal toda hora, outras recebendo alta e outras indo para a UTI* [...] *uma sensação muito ruim.* (M15)

[...] *não podia ter ninguém junto comigo e nem receber visita* [...] *eu só pegava ela de máscara, com o álcool e de máscara, 24h de máscara, até ela completar 15 dias* [...] *eu fiquei num quarto sozinha* [...] *foi a primeira bebê a nascer na ala do COVID* [...] *na primeira noite, antes dela nascer, tinha pessoas que chamavam as enfermeiras desesperadas, pessoas que tentavam respirar, que faziam um barulho que não sei explicar como se fosse puxando a respiração com muita força. Fiquei bem assustada* [...]. (M14)

*Não é fácil você ficar sozinha. Dá uma sensação de abandono* [...] *de você estar abandonada num lugar.* (M1)

A comunicação com a família era intermediada pelos profissionais, por ligação telefônica ou por ligação de vídeo ou de voz por aplicativo de celular. Permanecer com o telefone celular não foi permitido a todas as mulheres. As que ficaram privadas do uso do celular se sentiram mais sozinhas, abandonadas, com saudade da família.

Elas encontravam alguma tranquilidade e conforto emocional mediante contato por celular, por ligação de voz ou de vídeo com a família, e quando podiam ver o filho, mesmo que por vídeo chamada, intermediada pelos profissionais de saúde, *a priori,* assistente social e médico.

[...] *faziam vídeos dele lá* [do RN] *eu sabia que ele estava bem cuidado, isso me confortava um pouco* [...]. (M8)

*Pelo celular, chamada de vídeo* [...], *quando eu não fazia ligação de vídeo* [...] *o médico da COVID, ele pegava o celular, acho que do hospital, ou dele, não sei, fazia chamada de vídeo, explicava meu quadro, mostrava a neném, que a neném estava bem.* (M7)

*Era assistente social* [que falava com a família], *porque o meu celular, quando eu fiquei entubada, eles colocaram as minhas coisas numa sacola para dar para minha mãe.* [...] *Assim, a saudade apertava, meu coração doía, me sentia sozinha.* (M12)

## DISCUSSÃO

Em razão de as gestantes possuírem um sistema imunitário suprimido, confirma-se o maior risco de desenvolverem doenças graves ou críticas associadas à COVID-19, em particular pneumonia e insuficiência respiratória, condicionando à internação e antecipação do parto, além de maior risco de ruptura prematura de membranas, parto prematuro, aborto espontâneo, pré-eclâmpsia e parto cesáreo^(9-11)^. Quanto aos desfechos perinatais, sustenta-se o risco aumentado de sofrimento fetal, apgar <7 no quinto minuto, asfixia neonatal, baixo peso ao nascer, internação em UTI neonatal, óbito perinatal^(9,10)^.

Diante desses dados clínicos e das experiências das mulheres, a pandemia revela a complexidade e a fragilidade humana em meio às incertezas, às situações desconhecidas e inesperadas, relacionadas à gestação, ao parto e ao nascimento. Essas condições estão atreladas aos desafios existenciais, que revelam as forças e as fraquezas do ser humano, aos controles e aos descontroles sob a vida, ao futuro incerto e à estreita relação dos seres humanos com a morte^(2)^.

O nascimento é muito expectado pelas famílias, mas não o pré-termo, de modo inesperado, cuja internação em UTI neonatal, muitas vezes, é requerida. Assim, o RN, quando separado dos pais, afeta-os emocionalmente durante toda a internação, podendo desencadear emoções negativas, especialmente na mãe^(12)^. Essa condição intensifica pelas restrições da pandemia^(3)^ que, em alguns casos, nem um nem outro genitor puderam ver, tocar ou estar com o RN. Esses fatores agravaram os já elevados níveis de sofrimento das mulheres^(6,13)^.

Estudo global, com 2.103 participantes, de 56 países, aponta que 52% dos entrevistados não foram autorizados a ter outra pessoa presente durante o nascimento. O aumento das percentagens se relacionou às restrições no país de residência dos inquiridos. Ainda, 21% dos respondentes indicaram que ninguém estava autorizado a estar junto com o bebê na UTI. A frequência e a duração da presença permitida dependiam da extensão das restrições^(14)^.

A internação do RN em ambiente intensivo e as restrições de acesso dos pais devido à COVID-19, consequentemente, geraram um obstáculo para o vínculo precoce entre mãe/pai e filho, elevaram a solidão e o isolamento das mães que estavam com a infecção. Logo, as medidas de proteção inerentes à pandemia se mostraram adversas aos pais^(15)^.

Entre as adversidades, pode-se apontar o comprometimento do aleitamento materno exclusivo no pós-parto, identificado pelos relatos das mulheres, relacionado ao afastamento dos RN de suas mães e à autoeficácia na amamentação. Assim, fatores emocionais da mãe, comprometimento do vínculo dela com o RN e a falta de apoio social podem ter interferido no aleitamento materno^(16)^.

Conforme estudos abrangentes, importante parcela de mulheres que experienciaram o parto na vigência da pandemia de COVID-19 apresentou impacto negativo na saúde mental perinatal, vivenciando depressão, tristeza, ansiedade, insegurança e sintomas comórbidos, e a condição emocional das mulheres pode condicionar danos à amamentação^(4,6,13)^.

Apesar das dificuldades experienciadas, algumas mulheres conseguiram manter o aleitamento materno de seus filhos, com apoio de profissionais de enfermagem do Banco de Leite Humano (BLH). Comprova-se a importância deste serviço para proteção, promoção e apoio à amamentação, particularmente para mães de bebês que não podem mamar no peito pela prematuridade^(17)^.

O leite de doadoras se faz importante quando a mãe de pré-termos tem baixa produção láctea ou ausência de produção. O aumento dos níveis de educação em saúde sobre o funcionamento e a importância do BLH pode ter impacto positivo na captação dessas doadoras^(18)^.

No parto, nascimento e pós-parto, diante da necessidade de reduzir riscos de transmissão viral, direitos da mulher deixaram de ser prioridade, e passou-se a seguir recomendações científicas em voga, como reduzir a circulação de pessoas, avaliar a necessidade de interrupção da gestação, preferir a anestesia peridural à geral, estimular a amamentação, embora fosse contraindicado o contato pele a pele e indicado manter a mulher isolada do RN^(11)^. Algumas, inclusive, são contraditórias.

Pelo paradigma da complexidade^(2,7)^, o contraditório, a ordem e a desordem, o equilíbrio e o desequilíbrio, o caos e o adverso fazem parte das experiências cotidianas. Em meio a isso, na pandemia, os profissionais de saúde mantiveram suas atividades, expostos ao contágio e à morte, como missionários. Mantiveram as interações e interrelações para a assistência obstétrica no parto e pós-parto, adaptando-se aos modos de cuidar. Entre os profissionais, alguns demonstraram atitudes essencialmente humanas e solidárias, mas outros praticaram atos contrários ao cuidado humano esperado.

Essas situações, embora experienciadas em cenário em desordem, devido à pandemia, estão atreladas à ação humana, que demonstraram desconsideração pelo outro - a mulher e o RN. Um movimento contraditório ao cuidado humano, um momento complexo não compreendido por todos os profissionais que cuidam, com recomendações de assistência ao parto que não foram seguidas.

Estudos confirmam antagonismos na assistência. De um lado, mulheres que tiveram satisfação com o atendimento pelos profissionais. Estes, por meio de atitudes e palavras, promoveram conforto, tranquilidade, alívio da dor, bem-estar, respeito à autonomia e valorização da mulher durante o processo parturitivo^(19)^. Praticaram um cuidado sensível e empático, em meio ao aumento da demanda de trabalho e medo da contaminação viral^(4)^.

Do outro lado, mulheres experienciaram sentimentos de abandono, desconforto, negligência do cuidado, com relações verticalizadas entre mulher e profissional^(19)^, particularmente na escolha da via de parto^(4)^. Diante disso, acrescentou-se uma exposição desnecessária às mulheres e aos neonatos, com riscos de desfechos inesperados^(19)^, intensificados pela infecção por COVID-19^(9)^.

Essas condições configuram retrocessos na assistência ao parto. Sobre a atuação dos profissionais envolvidos no processo de parturição, aponta-se a importância do atendimento das necessidades humano-afetivas das mulheres, além das necessidades técnicas relacionadas ao parto e nascimento. Afirma-se a necessidade de as mulheres serem consideradas como um ser uno e múltiplo, com suas fragilidades, medos e incertezas inerentes ao momento experienciado. Deixar uma parturiente sozinha ou decidir estar junto implica ato de solidariedade e humanidade inerente aos seres humanos^(2,7)^, atitude esperada de profissionais de saúde responsáveis pelo cuidado.

No Brasil, o direito à presença de acompanhante no parto é respaldado pela Lei Federal nº 11.108 desde 2005^(20)^. Embora houvesse recomendação para ser mantido o acompanhamento da mulher durante a pandemia^(11)^, a sua exclusão teve a intenção de promover segurança a elas, aos bebês e profissionais de saúde^(3)^. Essa condição fere esse direito e causou impacto emocional às mulheres. Convém salientar que, em 2023, foi publicada a Lei n**º** 14.737, que garante à mulher acompanhamento por profissional de saúde indicado pela instituição; se a mulher não indicar acompanhante, no caso de sedação ou rebaixamento do nível de consciência ou em atendimento em centro cirúrgico^(21)^.

O plano de parto e nascimento, que expressa as preferências, desejos e expectativas da mulher em relação ao parto/nascimento^(22)^ possui indiscutível relevância na efetividade da dignidade humana, do direito à saúde e à integridade física e psíquica da mulher em processo parturitivo^(23)^. É positivo também para cesarianas programadas, tendo em vista que todas as mulheres, independentemente da via de parto, podem receber cuidados respeitosos, conforme desejam^(24)^. Destarte, ignorar o plano de parto e nascimento ou o desejo da mulher implica praticar atos contra a vontade dela^(23)^. Entretanto, a maioria das mulheres desconhece o plano de parto e nascimento^(25)^ e não exerce sua autonomia.

Acrescenta-se que as mulheres acometidas por COVID-19 e que desejavam parto vaginal poderiam tê-lo, desde que as avaliações clínica e obstétrica fossem favoráveis, cabendo interrupção cirúrgica da gestação quando as avaliações fossem desfavoráveis e/ou houvesse alterações na vitalidade fetal^(11)^. Assim, mesmo que a vontade da mulher não esteja expressa em um documento, faz-se necessário respeitar o que é por ela desejado^(24)^, considerando a ausência de riscos clínicos e ou obstétricos, no parto e nascimento^(11)^.

No entanto, a autonomia da mulher depende de condições culturais e sociais. A mulher é capaz de fazer escolhas, de tomar decisões, de examinar condutas, com autonomia e liberdade, mas também está sujeita à heteronomia, pois o atendimento das suas escolhas depende dos profissionais que lhe assistem e de outras condições sobre as quais ela não tem controle. Nesta lógica, a mulher pode ter a impressão de ser livre em suas escolhas, sem ser^(7)^.

O isolamento em ambiente restrito a pessoas com COVID-19 (enfermaria ou UTI), sem acompanhante, com necessidade de uso de máscara (embora algumas se recusaram a usar), provocou sofrimento às mulheres^(4,26)^. O fato de serem assistidas por profissionais que nem sempre eram os que tinham formação ou capacitação em obstetrícia criou uma barreira e comprometeu a assistência.

Devido à ausência do acompanhante no período parturitivo e do isolamento, para suporte emocional às mulheres, profissionais de saúde utilizaram estratégias de aproximação delas com a família, por meio de chamadas de vídeo do celular^(4)^. Destaca-se a necessidade de maior apoio àquelas que apresentavam sofrimento e consequente comprometimento da saúde mental perinatal^(6,13)^.

Embora o atendimento psicológico para as mulheres que experienciaram o parto, nascimento e puerpério na vigência da COVID-19 fosse recomendado para minimizar o sofrimento vivenciado^(26)^, não se constatou isso pelos depoimentos. Profissionais de enfermagem e de medicina foram os que principalmente buscavam abrandar os desafios das experiências das mulheres, oferecendo apoio emocional, seguidos das assistentes sociais, que intermediavam o contato com a família.

### Limitações do estudo

O estudo teve como limitação ter realizado entrevistas com mulheres que experienciaram parto e nascimento em um único hospital, todavia referência para parto de mulheres com COVID-19.

### Contribuições para as áreas da enfermagem, saúde ou políticas públicas

O estudo contribui com reflexões acerca da assistência materna, à luz da complexidade, em contextos inesperados, de incertezas e contradições, como o da pandemia de COVID-19.

## CONSIDERAÇÕES FINAIS

Compreendeu-se que a experiência de parto/nascimento/pós-parto foi diferente do que as mulheres esperavam, permeada de imprevisibilidades e de incertezas, intensa em emoções e significados que causaram sofrimento às mulheres. As restrições de contato e a ausência do acompanhante comprometeram a saúde mental delas, os cuidados do RN e o autocuidado no puerpério. O vínculo com o RN e a amamentação foram comprometidos.

Alguma tranquilidade e conforto emocional puderam ser experienciados pelas mulheres quando podiam ter contato com a família ou ver o RN pré-termo (que estava em UTI neonatal), mediante ligação de voz ou de vídeo, comumente intermediada por profissionais de saúde.

No que diz respeito à experiência da assistência, notaram-se condições antagônicas. Enquanto alguns profissionais de saúde demonstraram atitudes de cuidado humano, outros não o fizeram. Identificaram-se retrocessos de práticas consoantes à atenção obstétrica.

Recomenda-se respeito aos direitos das mulheres já conquistados na assistência ao parto, assegurados por lei, como a presença de acompanhante, bem como o cumprimento das recomendações obstétricas vigentes na assistência ao parto e nascimento, designação de profissionais capacitados e imbuídos de cuidado para assistir a mulher e o RN em alojamento conjunto e acompanhamento de mulheres que experienciaram o parto na vigência do diagnóstico da COVID-19, no aspecto da saúde mental, por profissionais da Atenção Primária à Saúde.
